# Opening a window into the riddle of misophonia, sensory over-responsiveness, and pain

**DOI:** 10.3389/fnins.2022.907585

**Published:** 2022-08-03

**Authors:** Adi Efraim Kaufman, Irit Weissman-Fogel, M. Zachary Rosenthal, Ricky Kaplan Neeman, Tami Bar-Shalita

**Affiliations:** ^1^Department of Occupational Therapy, School of Health Professions, Faculty of Medicine, Tel Aviv University, Tel Aviv, Israel; ^2^Physical Therapy Department, Faculty of Social Welfare and Health Sciences, University of Haifa, Haifa, Israel; ^3^Department of Psychiatry and Behavioral Sciences, Center for Misophonia and Emotion Regulation, Duke University Medical Center, Durham, NC, United States; ^4^Department of Communication Disorders, School of Health Professions, Sackler Faculty of Medicine, Tel Aviv University, Tel Aviv, Israel

**Keywords:** sensory over-responsiveness, pain sensitivity, misophonia, sensory processing, ecological sounds, auditory hyperalgesia, auditory analgesia

## Abstract

**Introduction:**

Misophonia and sensory over-responsiveness (SOR) share physiological and psychological symptoms. While individuals with SOR demonstrate pain perception alterations, these were not explored in misophonia.

**Methods:**

This exploratory study comprised thirty healthy adults with (*n* = 15; based on the Misophonia Questionnaire) and without misophonia. The Sensory Responsiveness Questionnaire (SRQ) was used for evaluating sensory responsiveness. In addition, psychophysical tests were applied for quantification of: (i) stimulus-response function of painful stimuli, (ii) the individual perceived pain intensity, (iii) pain modulation efficiency, (iv) auditory intensity discrimination capability, and (v) painful and unpleasantness responses to six ecological daily sounds using the Battery of Aversiveness to Sounds (BAS).

**Results:**

Individuals with misophonia reported higher scores in the SRQ-Aversive (*p* = 0.022) and SRQ-Hedonic (*p* = 0.029) scales as well as in auditory (*p* = 0.042) and smell (*p* = 0.006) sub-scales, indicating higher sensory responsiveness. Yet they were not identified with the SOR type of sensory modulation dysfunction. Groups did not differ in the pain psychophysical tests, and in auditory discrimination test scores (*p* > 0.05). However, in the misophonia group the BAS evoked higher pain intensity (*p* = 0.046) and unpleasantness (*p* <0.001) ratings in the apple biting sound, and higher unpleasantness rating in the scraping a dish sound (*p* = 0.007), compared to the comparison group.

**Conclusion:**

Findings indicate increased sensory responsiveness in individuals with misophonia, yet not defined as SOR. Thus, this suggests that misophonia and SOR are two distinct conditions, differing in their behavioral responses to painful and non-painful stimuli.

## Background

The recently published consensus definition of misophonia (Swedo et al., 2021) defines misophonia as “a disorder of decreased tolerance to specific sounds or stimuli associated with such sounds” (p. 22). These aversive sensory stimuli, commonly named misophonia triggers, are expressed physiologically (Edelstein et al., 2013; Johnson et al., 2013; Brout et al., 2018), severely impact daily function and social participation (Edelstein et al., 2013; Wu et al., 2014; Zhou et al., 2017; Kumar et al., 2021; Swedo et al., 2021), and are suggested to contribute to mental health difficulties (Schröder et al., 2013; Erfanian et al., 2019; Swedo et al., 2021). Indeed, misophonia has been reported to co-occur with psychiatric or neurological conditions (e.g., mental health disorders, attention deficit hyperactive disorder) (Cusack et al., 2018; McKay et al., 2018; Rouw and Erfanian, 2018; Erfanian et al., 2019; Swedo et al., 2021; Siepsiak et al., 2022), indicating that whether or not misophonia is a disorder in its own right is yet to be determined empirically (Swedo et al., 2021). Thus, research examining the nature and features of misophonia is needed to better characterize and differentiate this disorder (Swedo et al., 2021).

Neuroticism is a trait associated with misophonia (Cassiello-Robbins et al., 2020; Jager et al., 2020; Guetta et al., 2022) [i.e., moody, anxious, and tense (Goldberg, 1990)]. It is manifested in misophonia as behavioral and psychological responses to misophonia triggers including irritation, anger, anxiety, disgust, general psychological distress, and difficult regulating emotions (Rouw and Erfanian, 2018; Swedo et al., 2021). Like misophonia, sensory over-responsiveness (SOR) has been widely reported to co-occur with negative emotionality and psychological distress (Kinnealey et al., 2011; Bar-Shalita and Cermak, 2016; Carpenter et al., 2019) which significantly interferes with everyday function and quality of life (Cosbey et al., 2010; Carter et al., 2011; Kinnealey et al., 2011; Bar-Shalita et al., 2015; Bar-Shalita and Cermak, 2016). Unlike misophonia, characterized by hyper-sensitivity mainly in the auditory modality, specifically to human sounds, yet not solely (i.e., olfaction) (Brout et al., 2018; Swedo et al., 2021), SOR is characterized by multi-modal sensory hyper-sensitivity (Zero, 2005; PDM, 2006; Miller et al., 2007; Interdisciplinary Council on Developmental and Learning Disorders, 2012). SOR, a type of sensory modulation dysfunction, alters the ability to regulate behavioral adaptive responses to everyday sensory stimuli, in one or more sensory modalities (Miller et al., 2007). Specifically, individuals with SOR perceive non-painful daily stimuli as unpleasant and painful, lasting longer compared to non-SOR individuals (Kinnealey et al., 2011; Bar-Shalita et al., 2015). Likewise, laboratory testing of experimental pain in individuals with SOR who are otherwise healthy, utilizing psychophysical pain paradigms, indicated hyperalgesia (enhanced pain intensity) which lasted longer compared to controls (Bar-Shalita et al., 2009b,^2011^, ^2014^; Weissman-Fogel et al., 2018). Moreover, our research found that SOR and the personality trait of neuroticism together contribute to enhanced pain sensitivity to daily sensations, experienced as more aversive by individuals with SOR, compared to healthy controls (Bar-Shalita and Cermak, 2020). Given sparse reports on sensory hyper-sensitivity in other modalities beyond audition in individuals with misophonia (Edelstein et al., 2013; Wu et al., 2014; Schröder et al., 2019), and since pain perception has not been reported in misophonia, it is worthy to study SOR and pain perception in individuals with misophonia. Taken together, misophonia and SOR share symptoms anchored in the pattern of reacting to sensations cued by environmental stimuli, eliciting suffering, and functional limitations. Accordingly, it is somewhat surprising that the relationship between misophonia and SOR has yet to be rigorously empirically tested. Because (a) misophonia triggers are perceived as aversive sensations, (b) pain hypersensitivity is linked to SOR, and (c) misophonia may be conceptualized as a phenomena associated with SOR, the primary aim of the present study was to examine the relationships among misophonia, SOR, and pain hypersensitivity. Specifically, we hypothesized that (1) misophonia and SOR will be positively correlated, and (2) individuals with misophonia will demonstrate pain hypersensitivity compared to healthy controls using quantitative sensory testing (QST) and self-report measures.

## Materials and methods

This exploratory research was approved by the review committee at Tel Aviv University and the Helsinki Committee at Sheba Medical Center (5360-18-SMC). All participants completed and signed an informed consent form before enrolling in the study.

### Participants

A non-referred convenience sample of thirty healthy adults aged 18–50 years (73% female, *n* = 22; *M* age 30.5 years, *SD* = 9.84), with (*n* = 15, study group) and without misophonia participated in this study. Individuals with self-identified misophonia were recruited *via* misophonia social networks online, and healthy individuals (*n* = 15, comparison group) recruited through a pool of individuals interested in participating in research. Exclusion criteria stipulated audiological (hearing loss, hyperacusis, and tinnitus or other) neurological, psychiatric, developmental, or chronic pain diagnoses, and language proficiency. Exclusion criteria included the use of analgesia or consumption of psychoactive substances less than 24 or 6 h, respectively, before arriving at the lab. The study group inclusion reported a score of 7≤ on the impairment rating scale of the Misophonia Questionnaire (MQ) (Wu et al., 2014) (see below). The comparison group inclusion criteria included scoring <6 on the MQ (Wu et al., 2014) as well as scoring within the normal cut-off scores on the Sensory Responsiveness Questionnaire-Intensity Scale (SRQ-IS) (Bar-Shalita et al., 2009a) (mean ± 1 SD) [SRQ-Hedonic <2.43; SRQ-Aversive <2.13], demonstrating no sensory modulation dysfunction.

### Measures

#### Self-report questionnaires

Misophonia was assessed using the *MQ* (Wu et al., 2014), a three-part self-report questionnaire aimed at assessing misophonia consisting of: (1) *Misophonia Symptom Scale* which examines the presence of specific sound sensitivities (e.g., eating, tapping, throat sounds); (2) *Misophonia Emotions and Behaviors Scale* which examines emotional and behavioral reactions associated with misophonia, and (3) *Misophonia Severity Scale* which was adapted from the National Institute of Mental Health Global Obsessive-Compulsive Scale (Murphy et al., 1982) applicable for misophonia utilizing a 15 point rating scale (1 “low sensitivity” up to 15 “severe sensitivity”). A score equal or greater than 7 indicates clinically significant “moderate sound sensitivities”that cause “significant interference” (Wu et al., 2014). High internal reliability (Cronbach’s α = 0.88−0.90), convergent and distinct validity were reported (Wu et al., 2014). In this study we used the Misophonia Severity Scale.

Sensory responsiveness was assessed using the *SRQ-IS* (Bar-Shalita et al., 2009a), a self-report questionnaire assessing behavioral response patterns to daily sensation, and aimed to identify sensory modulation dysfunction in adolescents and adults. The questionnaire consists of 58 statements describing everyday situations involving stimulation in one of the following modalities: auditory, visual, gustatory, olfactory, vestibular, and somatosensory, excluding pain. Participants rate the intensity of the enjoyment or disturbance in the situation described in each statement using a five-point Likert scale (1 “not at all” up to 5 “very”), comprising 2 scales: Applying the SRQ-Aversive scale (32 items), scores higher than the normal mean cut-off score (+2 *SD*; 1.87 + 0.52) indicate SOR. Applying the SRQ-Hedonic scale (26 items), scores higher than the normal mean cut-off score (+2 *SD*; 2.10 + 0.66) indicate sensory under responsiveness (SUR) (Weissman-Fogel et al., 2017). Internal reliability (Cronbach’s α = 0.90−0.93), and test–retest reliability (*r* = 0.71−0.84; *p* < 0.001−0.005) as well as content, criterion, and construct validity were reported (Bar-Shalita et al., 2009a).

Daily pain sensitivity was assessed using the *Pain Sensitivity Questionnaire (PSQ)* (Ruscheweyh et al., 2009), a self-report questionnaire, aimed at assessing the intensity of daily pain sensitivity. The PSQ contains 17 items describing everyday situations associated with a wide range of somatosensory pain. Fourteen of the items relate to situations that describe painful situations for most people (e.g., hot, cold, sharp, or dull). The three remaining items (5, 9, and 13) describe situations typically not rated as painful by healthy participants (e.g., taking a warm shower). Participants are requested to imagine how painful this situation would be for them and use a 10 point response scale ranging from 0 (not painful at all) to 10 (worst pain imaginable). The questionnaire provides a total score (PSQ-total) and two additional scores (PSQ-moderate, PSQ-minor) ranging from 0 to 10; higher score denotes high sensitivity to daily pain. The PSQ has internal reliability for the total score (α = 0.92), as well as for the 2 sub-scales PSQ-minor: α = 0.81; PSQ-moderate α = 0.91, and test–retest reliability (ICCs = total score 0.83; PSQ-moderate 0.79; PSQ-minor 0.86). Content, criterion, and construct validity have been previously reported for this measure (Ruscheweyh et al., 2012).

#### Pain psychophysics evaluation applying quantitative sensory testing

Prior to testing, participants were informed that the heat stimuli will be delivered at intensities not causing harm or damage and are safe in line with the Food and Drug Administration (FDA) requirements. Heat stimulus, using a computerized thermal stimulator, the Pathway system for Contact Heat-Evoked Potential Stimulator (CHEPs) (Medoc Ltd. Advanced Medical Systems, Ramat Yishai, Israel), was applied on the central volar aspect of the right forearm using a flat disk probe 572.5 mm^2^ thermode. After each stimulus the thermode was removed to avoid adaptation/sensitization. During the inter-stimulus interval (ISI) periods participants were asked to rate the pain intensity after each stimulus using a verbal numeric pain scale (NPS; 0 = no pain at all; 100 = worst imaginable pain). The baseline temperature was 32°C, increased temperature rate was set on 70°C/sec, and decreased rate was set on 40°C/sec for all stimuli. Following familiarization with the pain stimuli and required rating the following tests were performed:

##### Dose response test

**FIGURE 1:**
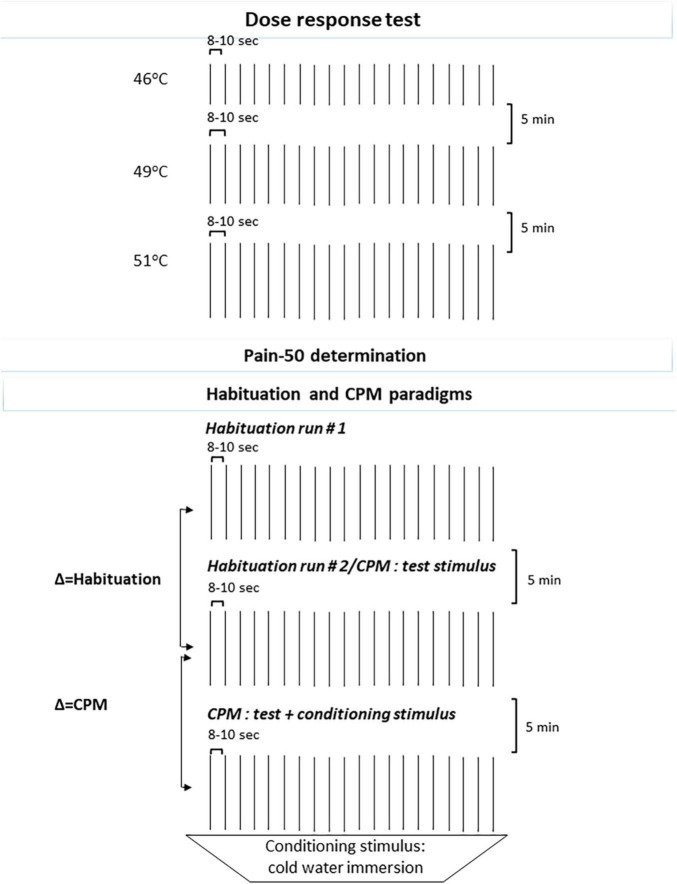
Schematic representation of the QST: *Dose response test* and *Habituation* (Runs 1 and 2) and *CPM* tests paradigms. QST, quantitative sensory testing; CPM, conditioned pain modulation.

Three separate runs, each comprising of 20 CHEPs heat stimuli (8–10 s ISI, randomized) at 46, 49, and 51°C were utilized, counterbalancing 46 and 49°C randomly to avoid risk of order effects. Between runs interval of 5-min was utilized (see Figure 1).

##### Determination of testing temperature

Since pain evoked from heat stimuli depends on the peripheral and central pain pathways function and varies among participants (Staud et al., 2004), the testing temperature for the following somatosensory psychophysical tests was individually tailored to evoke a peak pain magnitude of 50/100 (henceforth pain-50) on the NPS. Searching for the individual pain-50 temperature, we used the Methods of Levels. The initial temperature choice was based on the pain ratings each subject provided in the *Dose response* test (i.e., 46, 49, and 52°C). Thereafter, temperature search was respectively, decreased or increased by 1°C followed by increase/decrease of 0.5°C until reaching the desired pain level of 50. When 2 out of 3 CHEPs stimuli (ISI 8 s) were rated as 50 on the NPS, the individually tailored temperature of pain-50 was attained and served as the individual testing temperature. For participants not reporting 50 on the NPS, the maximum temperature (55°C) was set as their testing temperature (Weissman-Fogel et al., 2018).

##### Habituation paradigm

Two runs of 20 CHEPs heat stimuli each (ISI of 8–12 s) utilizing the individually tailored pain-50 temperature were applied, with between runs interval of 5-min. Participants were asked to rate the pain intensity following each stimulus. A lower average in the second run indicated habituation (see Figure 1; Weissman-Fogel et al., 2018).

##### Conditioned pain modulation paradigm

Using the cold water tub (8–10°C) as a conditioning test stimulus, participants inserted their left hand and were requested to rate the pain intensity after 10 s. Thereafter, while the hand remained in the cold water tub, participants received a series of 15 heat stimuli (test stimuli) (ISI 8–12 s) delivered to the right forearm at the individually tailored pain-50 temperature and were asked to rate the pain intensity after each stimulus using the NPS. At the end of this series, participants reported their left hand pain intensity before removing their hand from the cold water tab. Conditioned Pain Modulation (CPM) magnitude was derived from the deduction of the mean pain intensity ratings of the test stimuli given alone from the mean pain intensity ratings given simultaneously with the conditioning stimulus. Negative values indicate an efficient CPM (see Figure 1; Yarnitsky et al., 2012).

#### Auditory psychophysics evaluation

Sounds were delivered to both ears *via* headphones Audio-Technica, Japan (ATH-M40×). Sounds were calibrated using an Audio Scan Verifit VF-1 (Etymonic Design Inc., Dorchester, ON, Canada) in a 2-cm^3^ HA2 coupler, by means of a manual control procedure, with an A-weighted filter. To eliminate tester bias we ensured no eye contact between the participant and the researcher.

##### Auditory intensity discrimination test

To ensure intact intensity discrimination, we measured auditory discrimination acuity by using a computerized test. Six intensity levels of 1-kHz tone (pure tone produced at a stable sound pressure level), differing in amplitude by increments of 5 dB (range = 60–85 dB), lasting for 2 s each (ISI 8 s), were delivered three times in a random order. Participants were asked to verbally rate the sound intensity on a computerized numerical scale ranging from least intense (Swedo et al., 2021) to most intense (Schröder et al., 2013). Before testing, participants were familiarized with the least and most intense sounds twice (Assayag et al., 2020).

##### Battery of aversiveness to sounds

Computerized testing with six ecological sounds each applied 3 times: (1) scraping a dish, (2) apple eating, (3) ticking clock, (4) water drops, (5) alarm, and (6) 1 kHz tone (a pure tone produced at a stable sound pressure level). A total of 18 sounds (each: 30 s duration; ISI 30 s) were delivered in- within and between participants random order. The sounds were calibrated to volume levels up to 80 dB SPL. Sounds 1–5 were normalized for intensity (78–80 dB SPL) using the Manual control mode of the Verifit VF-1 (Audioscan, 2006) analyzed by 1/12th octave, A-weighted filter, at a rate of 384 ms. After each sound, participants were instructed to verbally rate the pain and unpleasantness intensities (Price et al., 1983) on an 11-point scale (0 “no pain/no disturbance” up to 10 “maximum pain possible/the highest level of disturbance you can imagine “) (Mazor-Karsenty et al., 2019; Assayag et al., 2020).

### Procedure

The study was administered in the Sensory Integration Lab at Tel Aviv University in a quiet, air-conditioned room (22–24°C) with ambient noise typically not exceeding 45 dB SPL and the participant sitting on a comfortable recliner. The session lasted for approximately 2 h. After verifying the inclusion criteria using the MQ, SRQ-IS, and demographic questionnaire, participants undertook the psychophysical testing in counterbalanced order. Thereafter, participants completed the PSQ electronically.

### Data analysis

Data analysis was performed using SPSS (Quintero et al., 2013) software version 27. Descriptive statistics were used to describe the population and study variables. The Shapiro–Wilks test was used to test the distribution type of the dependent variables. Group differences were examined *via* Mann–Whitney- or *t*-tests, according to variables distribution type. Pearson Correlation Coefficient or the Spearman’s Rank Coefficient tests were used to test correlations. Correlations were compared between the groups using Fisher’s z transformation test where they were then treated as normal random variables. To determine the relative contribution of the independent variables [SRQ, PSQ, and battery of aversiveness to sounds (BAS-R)] in predicting the dependent variable (MQ), two multiple regression models were established, one for each group; additionally we established a model for the whole sample. All statistical tests were two-sided and tested at a 5% level of significance. Nominal *p*-values are presented.

## Results

### Demographic characteristics

No statistically significant group differences were found in age [study vs. control groups Mean (SD): 31.7 (11.77) vs. 29.29 (7.67)], sex (women 73.33% both groups), years of education [study vs. control groups Mean (SD): 14.67 (1.91) vs. 14.47 (7.39)] and dominant hand. A statistically significant group difference was found in the MQ scores, score Mean (SD); ranges among the study and comparison groups were 8.47 (1.68); 7–13, and 2.27 (1.90); 0–6, respectively. Of note, 66% of participants in the study group scored 7–8 on this MQ.

### Group differences in the Sensory Responsiveness Questionnaire and Pain Sensitivity Questionnaire scores

Statistically significant group differences were found in both SRQ scores, showing higher scores (higher responsiveness) in the study group. Yet, within the study group the two SRQ scores (SRQ-Hedonic; SRQ-Aversive) were found below cut-offs, indicating no sensory modulation dysfunction (SMD). We also examined group differences in the mean scores in all sensory modalities. A statistically significant group difference was found in the auditory and the olfactory sub-scales demonstrating higher scores (higher responsiveness) in the study group (Table 1). Groups did not differ in the mean scores of the other sensory sub-scales (vision, taste, vestibular, and somatosensory) (Table 1).

**TABLE 1:** Group differences in the SRQ scores.

	Study group (*n* = 15)		Comparison group (*n* = 15)		*t/z*	*p*
		
	Median (IQR)	Mean (SD) (Range)	Median (IQR)	Mean (SD) (Range)		
SRQ-Aversive	2.03 (1.50–2.16)	(0.31) 1.91 (1.41–2.3)	1.70 (1.50–1.87)	1.67 (0.22) (1.08–2.11)	2.434	**0.022**
SRQ-Hedonic	1.96 (1.77–2.30)	2.02 (0.32) (1.56–2.7)	1.87 (1.38–2)	1.74 (0.34) (1.08–2.11)	2.309	**0.029**
SRQ-Auditory	2 (1.50–2.33)	1.96 (0.58) (0.83–3)	1.67 (1.33–1.83)	1.59 (0.34) (1–2.33)	2.136	**0.042**
SRQ-Olfactory	2.50 (2–3)	2.51 (0.76) (1.25–3.75)	1.83 (1.33–2.50)	1.63 (0.61) (1–3)	−2.770	**0.006**
SRQ-Visual	2 (1.83–2)	1.93 (0.37) (1.17–2.5)	1.83 (1.67–2.17)	1.84 (0.39) (1.17–2.67)	0.643	0.53
SRQ-Vestibular	2 (1.78–2.33)	2.04 (0.35) (1.33–2.56)	2 (1.67–2.22)	1.90 (0.38) (1.22–2.67)	1.006	0.32
SRQ-Somatosensory	1.71 (1.50–1.86)	1.67 (0.25) (1.09–2.05)	1.57 (1.45–1.67)	1.55 (0.2) (1.05–1.91)	1.404	0.17
SRQ-Taste	2.33 (2–2.50)	2.30 (0.41) (1.67–3.17)	1.83 (1.33–2.50)	1.93 (0.34) (1–3.33)	1.724	0.10

SRQ, sensory responsiveness questionnaire; IQR, interquartile range; SD, standard deviation. Bold values denote statistically significant group differences.

No statistically significant differences were found between the two groups in the PSQ scores (*p* > 0.05).

### Correlations of the Misophonia Questionnaire score with the Sensory Responsiveness Questionnaire and Pain Sensitivity Questionnaire scores

Within each of the groups no statistically significant correlations were found between the MQ and SRQ scores. Further, between group comparison in these correlations we found no statistically significant group differences were found. However, while in the comparison group the MQ significantly correlated with the PSQ total (*r* = 0.524, *p* = 0.04) and PSQ-Moderate (*r* = 0.525, *p* = 0.044) scores, no statistically significant correlations were found in the study group. Between group comparison in these correlations found no statistically significant group differences. Indeed, after running a bootstrap analysis we did not reach statistically significant correlations within the control group.

### Psychophysics

#### Group differences in thermal pain ratings

No statistically significant differences (*p* > 0.05) were found in pain intensity and unpleasantness ratings in Dose-Response, Testing Temperature (pain-50), Habituation, and CPM testing. Of note, the study group ratings were consistently slightly lower (Table 2).

**TABLE 2:** Group differences in thermal pain psychophysics tests ratings.

	Study group (*n* = 15)		Comparison group (*n* = 15)		*t/z*	*p*
		
	Median (IQR)	Mean (SD) (Range)	Median (IQR)	Mean (SD) (Range)		
Dose-Response 46°C	9.25 (5.8–12.5)	15.79 (19.37) (1.25–70)	12 (8.9–25.1)	19.11 (19.63) (0.45–76.45)	−0.850	0.395
Dose-Response 49°C	10.75 (8.4–23.8)	16.17 (14.04) (1.05–55.35)	16.6 (9.5–35.3)	23.30 (21.32) (0.75–84.35)	−0.100	0.319
Dose-Response 52°C	14.25 (9.8–21.5)	19.68 (19.84) (0.93–83.4)	21 (13.8–23.8)	25.21 (21.80) (0.8–87.45)	−1.26	0.206
Destination Temperature	55 (55–55)	54.63 (0.76) (53–55)	55 (53–55)	53.62 (3.49) (41.5–55)	−0.853	0.393
Habituation 1	17.3 (8.8–29.4)	20.68 (14.85) (0.55–55.5)	31 (12.0–40)	29.51 (17.67) (4.35–68.25)	−1.483	0.149
Habituation 2	21 (12–38.3)	22.69 (14.3) (0.51–48.25)	25 (17–44.3)	28.83 (17.12) (3.9–67.75)	−1.065	0.296
Conditioned pain modulation	−4 (−11.8 to –0.2)	−3.06 (14.01) (−23.05–29)	0.2 (−8–3.5)	−2.47 (10.54) (−26.9–17.8)	−0.730	0.943

SD, standard deviation; IQR, interquartile range.

#### Group differences in auditory discrimination

No statistically significant group differences were found (*p* > 0.05) in the Auditory Discrimination Test for all of the six intensity levels of the 1-kHz sound.

#### Group differences in the battery of aversiveness to sounds pain and unpleasantness ratings

Testing auditory pain intensity found statistically significant pain ratings only in the *apple eating* sound indicating higher ratings in the study group (Table 3). Testing ratings of unpleasantness found statistically significant group differences in *scraping a dish* and *apple eating* sounds, indicating higher ratings in the study group. No statistically significant group differences were found in pain intensity and unpleasantness ratings in the ticking clock, tone 1 kHz (Tone 1), water drops, and alarm sounds (Table 3).

**TABLE 3:** Group differences in auditory psychophysics pain and unpleasantness ratings.

		Study group (*n* = 15)		Comparison group (*n* = 15)		*t/z*	*p*
			
		Median (IQR)	Mean (SD) (Range)	Median (IQR)	Mean (SD) (Range)		
BAS-R Pain	Scraping a dish	0.67 (0–2.67)	2.53 (2.58) (0–10)	0.67 (0–2.67)	1.64 (2.33) (0–8)	−1.440	0.15
	Eating apple	0 (0–0.67)	2.35 (2.93) (0–8.33)	0 (0–0.67)	0.62 (1.25) (0–3.66)	−1.995	**0.046**
	Ticking clock	0 (0–0.67)	0.49 (1.13) (0–4)	0 (0–0.67)	0.47 (0.89) (0–2.66)	−0.364	0.71
	Tone 1 kHz (Tone 1)	0 (0–1.33)	1.73 (3.12) (0–10)	0 (0–1.33)	0.80 (1.26) (0–4)	−0.722	0.47
	Water drops	0 (0–0.33)	0.44 (1.38) (0–5.33)	0 (0–0.33)	0.20 (0.41) (0–1.33)	−0.336	0.74
	Alarm	0 (0–2)	1.55 (2.01) (0–7.33)	0 (0–2)	1.07 (1.68) (0–5.33)	−1.116	0.26
BAS-R unpleasantness	Scraping a dish	3.67 (2.3–5.3)	6.91 (2.29) (3–10)	3.67 (2.3–5.3)	4.22 (2.72) (0–10)	2.931	**0.007**
	Eating apple	3.67 (3.33–6)	8.49 (1.33) (5.33–10)	3.67 (3.33–6)	4.29 (2.3) (0–8.66)	−4.102	**<0.001**
	Ticking clock	3.33 (1–4.67)	2.58 (2.47) (0–8)	3.33 (1–4.67)	3.02 (2.28) (0–7.33)	−0.605	0.54
	Tone 1 kHz (Tone 1)	3.67 (1.67–6)	3.73 (2.31) (0.66–8)	3.67 (1.67–6)	3.73 (2.31) (0.66–8)	0.606	0.55
	Water drops	2.67 (1–3.33)	2.58 (1.97) (0–7)	2.67 (1–3.33)	2.58 (1.97) (0–7)	1.503	0.14
	Alarm	5 (1.67–6.33)	4 (2.47) (0–7)	5 (1.67–6.33)	4 (2.47) (0–7)	0.924	0.36

BAS-R, battery of aversiveness to sounds, ratings range 0–10; SD, standard deviation; IQR, interquartile range. Bold values denote statistically significant group differences.

### Correlations between the Misophonia Questionnaire and battery of aversiveness to sounds scores

No statistically significant correlations were found within each group. However, group comparisons indicated statistically significant group differences in the correlations between MQ scores and the unpleasantness ratings of the ticking clock *r* = −0.08 vs. 0.47 *p* = 0.039; water drops *r* = −0.22 vs. 0.49 *p* = 0.009; and alarm sounds *r* = −0.31 vs. 0.30, *p* = 0.028, study vs. comparison groups, respectively, showing negative low correlations in the study group, whereas positive low to moderate correlations were observed in the comparison group.

### The final multiple regression model to predict Misophonia Questionnaire

In the separate models for each group the residuals were not normally distributed based on the P-P plots, and the predictors were highly correlated showing a high multicollinearity (VIF > 10). Therefore, the results were not valid and we referred to the model on the whole sample.

In this model the residuals were normally distributed and showed homoscedasticity, yet high multicollinearity was identified. Since the main assumptions of this regression model had been met we refer to the results. The model was found statistically significant [*F*_(15,14)_ = 2.87; *p* = 0.03; *R*^2^ = 0.75], yet none of the effects i.e., SPQ, PSQ, and BAS-R were found statistically significant (*p* > 0.05).

## Discussion

This preliminary study is the first to test SOR and pain sensitivity using psychophysical measures *via* QST in individuals with misophonia. Using QST, our findings support the consensus definition (Swedo et al., 2021) by demonstrating that the sounds with differentially aversive responses in the misophonia group were mostly human-generated. This suggest a difference between SOR and misophonia, where SOR entails a very wide range of auditory stimuli that may be aversive, whereas misophonia may be associated, with some variability, with a more limited scope of aversive auditory cues. This conclusion is further supported by the absence of general sensory modulation dysfunction among individuals with misophonia in the study sample, namely, they were not defined having SOR. Yet, these individuals with misophonia scored higher in the normal range of multisensory responsiveness. Specifically they reported increased sensory responsiveness in the auditory and olfactory sensory systems, suggesting sensory responsiveness beyond the auditory system in misophonia. However, contrary to our hypothesis findings indicate that individuals with misophonia did not demonstrate pain hyper-sensitivity in different QST paradigms, but consistently reported lower pain ratings. This further suggest that misophonia is not similar to SOR.

The pain matrix includes brain areas processing of both noxious and non-noxious stimuli (Mouraux et al., 2011; Senkowski et al., 2014) suggestive of an interaction between sensory systems. Indeed, we have previously reported a coupling between multisensory systems and pain (e.g., Bar-Shalita et al., 2011, 2014, 2015, 2019; Weissman-Fogel et al., 2018; Granovsky et al., 2019), i.e., individuals with SOR demonstrate pain hyper-sensitivity in response to experimental and daily life pain stimuli. Further, chronic pain patients, e.g., fibromyalgia, temporomandibular disorders, and chronic pelvic pain show sensory hyper-sensitivity to non-noxious stimuli (Schrepf et al., 2018). Specifically to the auditory and pain systems, hyperacusis was found prevalent in chronic pain conditions (de Klaver et al., 2007; Irimia et al., 2008; Suhnan et al., 2017). Thus, based on the bidirectional shaping of the noxious and non-noxious sensory perception *via* painful and non-painful stimuli (Mouraux et al., 2011; Pomper et al., 2013; Senkowski et al., 2014), we speculated that the auditory aversive stimuli, at least those which may be considered triggers, will elicit pain response. Indeed, human sound, i.e., eating apple perceived not only as aversive for individuals with misophonia, but also as painful. This finding supports the auditory-pain interaction probably in cortical brain areas that have a role in multi-sensory integration such as S2, the insula, and the anterior cingulate cortex (Mouraux et al., 2011).

Contrary to our assumption, we did not find pain hyper-sensitivity in misophonia. This further supports the distinction between SOR and misophonia. Specifically, while in SOR abnormally intense central neural processing is the suggested mechanism (Parush et al., 2007; Zlotnik et al., 2015; Granovsky et al., 2019), increased activity and connectivity in top–down modulatory brain areas is evident in misophonia (Kumar et al., 2017). The latter may explain our finding that individuals with misophonia consistently rated lower pain intensities. In detail, the auditory and the pain systems share the same top–down modulatory mechanisms which involve prefrontal brain areas and parallel descending inhibitory components (Rauschecker et al., 2015; De Ridder and Vanneste, 2021). A key brain area in the descending inhibitory pathways is the periaqueductal gray that receives collaterals from the spinothalamic tract (Zhang et al., 1990) as well as form several auditory nuclei (Halladay and Blair, 2012; Wang et al., 2019), and have a role in auditory-induced analgesia (Dobek et al., 2014). Thus, we speculate that the prefrontal cortex which is part of a central “gatekeeping” system, evaluates the relevance and affective value of auditory stimuli and controls information flow including pain, *via* descending pathways, with an attempt to inhibit sensory stimuli. Indeed they successfully inhibit experimental pain stimuli and demonstrated efficient CPM, which evaluates the efficiency of the descending inhibitory pathways. The assumed excessive inhibitory processes in misophonia is also reflected in our findings demonstrating negative correlations between unpleasantness ratings of the ecological non-human sounds and the misophonia scale score in the misophonia group compared to controls who demonstrated opposite direction. These allude to a successful inhibition to auditory non-trigger sounds yet not to trigger sounds.

This is a preliminary study consisting of a small sample size. Further, we did not test the emotional aspect nor behavioral regulation profiles, both of which characterize misophonia and SOR, as well as affecting pain perception. Future studies should establish multiple regression models using independent variables that are not correlated, and use large samples. Further, future studies should investigate the link between somatosensory and auditory pain using neurophysiological tools.

To conclude, this preliminary study found increased sensory responsiveness in misophonia, yet not defined as SOR, and no differences in pain sensitivity. Thus, this suggests that misophonia and SOR are two distinct conditions, differing in their behavioral responses to painful and non-painful stimuli. Findings allude to future exploration of the pain, auditory analgesia, and auditory hyperalgesia neurophysiological mechanisms in misophonia (Manohar et al., 2020).

## Data availability statement

The original contributions presented in this study are included in the article/supplementary material, further inquiries can be directed to the corresponding author.

## Ethics statement

The studies involving human participants were reviewed and approved by Tel Aviv University and the Helsinki Committee at Sheba Medical Center (5360-18-SMC). The patients/participants provided their written informed consent to participate in this study.

## Author contributions

IW-F, MZR, and TB-S contributed to conception and design of the study. AE organized the database and performed the statistical analysis. AE and TB-S wrote first draft of the manuscript. IW-F, MZR, RK, and TB-S wrote sections of the manuscript. All authors contributed to manuscript revision, read, and approved the submitted version.
